# Nutrition as a systems regulator of brain aging trajectories

**DOI:** 10.3389/fnmol.2026.1825800

**Published:** 2026-04-21

**Authors:** Ludmila Müller, Svetlana Di Benedetto, Viktor Müller

**Affiliations:** Max Planck Institute for Human Development, Center for Lifespan Psychology, Berlin, Germany

**Keywords:** brain aging, epigenetic regulation, glial activation, gut-brain axis, metabolic reprogramming, neuroinflammation, nutritional neuroscience, precision nutrition

## Abstract

Nutrition is increasingly recognized as a central determinant of brain health across the lifespan. Beyond their classical roles as energetic substrates, dietary components and their bioactive metabolites may act as signaling molecules capable of reshaping neuronal and glial phenotypes through integrated metabolic, epigenetic, and immunological mechanisms. Emerging evidence positions nutritional inputs as dynamic regulators of synaptic integrity, cellular bioenergetics, neurotransmission, neuroimmune interactions, and blood–brain barrier function. These effects occur across multiple temporal and spatial scales, from acute modulation of neuronal excitability to long-term reprogramming of gene expression and chromatin landscapes. This mini-review integrates current molecular neuroscience perspectives to propose a systems-level framework in which nutritional signals act across interconnected regulatory layers linking peripheral metabolism with central nervous system homeostasis. We examine nutrient-sensing pathways that preserve proteostasis and synaptic resilience, as well as metabolic and membrane-associated processes that govern neuronal excitability, network stability, and mitochondrial quality control. Furthermore, we discuss how dietary modulation may influence glial activation states, neuroinflammatory cascades, and epigenetic remodeling, and how gut-derived metabolites contribute to these processes. Understanding nutrition as an active signaling network rather than a passive support system may offer novel opportunities for preventive and therapeutic intervention in neurodegenerative disorders such as Alzheimer’s disease and Parkinson’s disease, as well as in age-associated cognitive decline. We propose that targeted nutritional modulation represents a tractable strategy to reprogram brain aging trajectories toward enhanced resilience, functional plasticity, and long-term cognitive health.

## 1 Introduction

The preservation of brain health across the lifespan represents one of the central biomedical challenges of the twenty-first century. Increasing life expectancy has been accompanied by a rising prevalence of neurodegenerative disorders, including Alzheimer’s disease (AD) and Parkinson’s disease (PD), as well as age-associated cognitive decline that emerges even in the absence of overt pathology. While genetic susceptibility contributes to disease risk, converging lines of evidence suggest that environmental and lifestyle factors–particularly nutrition–may play decisive roles in shaping trajectories of brain aging, vulnerability, and resilience (Di Benedetto and Müller, 2019; Fang et al., 2026; Nourazarain and Vaziri, 2025; Polom and Boccardi, 2025).

Traditionally, nutrition has been viewed primarily through the lens of energy supply and structural maintenance. Within this classical framework, dietary components support neuronal function by providing substrates required for ATP production, membrane synthesis, and neurotransmitter biosynthesis. However, advances in molecular neuroscience, immunology, and systems biology now challenge this reductionist perspective. Nutrients and their bioactive metabolites are increasingly recognized as signaling entities capable of regulating gene expression, cellular metabolism, and intercellular communication across multiple biological scales. Rather than acting as passive inputs, nutritional signals dynamically interact with cellular sensing systems that coordinate metabolic adaptation, immune activity, and neural network stability (Fang et al., 2026; Goul et al., 2023; Zwilling et al., 2024).

The central nervous system (CNS) can be conceptualized as a dynamic cellular ecosystem composed of neurons, astrocytes, microglia, endothelial cells, peripheral immune components, and microbiota-derived influences. Brain function may therefore emerge from continuous cross-talk between metabolic and immune networks that extend well beyond the brain itself (Müller and Di Benedetto, 2025c; Müller et al., 2025c). Nutritional states appear capable of influencing this integrated system through nutrient-sensitive pathways regulating mitochondrial function, proteostasis, synaptic remodeling, and inflammatory tone. Importantly, many of these processes overlap with molecular mechanisms implicated in neurodegeneration, suggesting that disruptions in metabolic signaling and immune regulation may represent early drivers rather than late consequences of disease (Di Benedetto and Müller, 2019; Di Benedetto et al., 2017; Fiorito et al., 2025; Müller and Di Benedetto, 2025c).

A growing body of research further highlights the role of gut-derived metabolites as mediators linking peripheral nutrition with CNS function. Microbial transformation of dietary substrates generates bioactive molecules capable of modulating blood–brain barrier (BBB) integrity, neuroimmune interactions, and epigenetic regulation within neural cells. These findings support a paradigm in which the gut–brain axis may act as a distributed biochemical communication network translating nutritional exposure into long-term neural outcomes (Bano et al., 2024; Chenghan et al., 2025; Decarie-Spain et al., 2024; Krishaa et al., 2023; Müller and Di Benedetto, 2025a; Peter and Strober, 2023; Seo and Holtzman, 2024).

In parallel, emerging evidence demonstrates that metabolic states directly influence epigenetic programs that determine cellular identity and functional phenotypes. Nutrient availability may regulate chromatin remodeling, DNA methylation, and histone modifications, thereby shaping neuronal plasticity and glial activation states. Such mechanisms provide a plausible biological substrate through which lifelong nutritional experiences may accumulate into durable effects on cognitive aging and disease susceptibility (Bekdash, 2024; Missong et al., 2024; Perlmutter et al., 2024; Zhang et al., 2025).

Within this evolving framework, neurodegenerative disorders can be conceptualized not solely as neuron-centric pathologies but as systems-level failures involving disrupted metabolic coordination, chronic neuroinflammation, impaired cellular stress responses, and loss of network resilience (Müller and Di Benedetto, 2025c,^2026^; Müller et al., 2025d). Nutritional interventions may represent a practical and accessible strategy to influence metabolic, immune, and epigenetic pathways concurrently, with emerging evidence from human studies suggesting potential benefits for brain health and cognitive function (Tingö et al., 2024; Yi et al., 2025; Zwilling et al., 2024).

In this mini-review, we adopt a molecular neuroscience perspective to conceptualize nutrition as a dynamic regulator of brain resilience. Rather than recapitulating the extensive mechanistic detail already synthesized in recent systematic reviews, we intentionally prioritize the development of an integrative conceptual framework that bridges key processes across traditionally distinct domains. Drawing on insights from metabolism, neuroimmunology, and systems biology, we articulate how nutrient sensing, cellular bioenergetics, neuroimmune regulation, microbiome signaling, and epigenetic programming converge within a unified, systems-level model that shapes brain aging trajectories.

## 2 Nutrition as a neurobiological signaling system

The emerging recognition that nutrition may actively shape brain function requires a conceptual shift from substrate-centered views toward an integrative signaling paradigm. The CNS does not merely consume nutrients to sustain energetic demands; rather, it continuously interprets nutritional inputs as regulatory signals that coordinate cellular adaptation, intercellular communication, and network stability. Within this framework, dietary components and their metabolites may function as dynamic modulators of biological programs that can influence brain resilience across the lifespan (Aminian-Dehkordi et al., 2025; Charbit et al., 2025).

At the molecular level, nutrient availability is monitored by evolutionarily conserved sensing systems that integrate information about energy status, redox balance, and metabolite flux. Key regulatory hubs–including AMP-activated protein kinase (AMPK), mammalian target of rapamycin (mTOR), insulin/IGF signaling pathways, and nicotinamide adenine dinucleotide (NAD^+^)-dependent sirtuins–translate metabolic cues into transcriptional, epigenetic, and post-translational responses. These pathways collectively govern anabolic–catabolic balance, mitochondrial quality control, autophagy, and cellular stress resistance. Importantly, many of these nutrient-sensitive mechanisms overlap with pathways disrupted in neurodegenerative disorders, suggesting that impaired metabolic signaling may represent an early systems failure preceding overt neuronal loss (Bonkowski and Sinclair, 2016; Goul et al., 2023; Lee et al., 2021; Lin et al., 2023; Thakur et al., 2023; Vergara Nieto et al., 2025).

The brain operates as a metabolically heterogeneous organ in which neurons, astrocytes, microglia, oligodendrocytes, and vascular cells fulfill specialized yet interdependent roles (Beard et al., 2021; Müller et al., 2025a,d; Pang et al., 2026). Nutritional signals therefore propagate through cellular networks rather than acting on isolated cell types. Astrocytes regulate metabolic substrate allocation and antioxidant capacity; microglia adjust immune surveillance and inflammatory tone according to metabolic conditions; endothelial cells adapt barrier permeability in response to circulating metabolites. Consequently, dietary states can influence not only intracellular metabolism but also the emergent properties of neural circuits, including synaptic plasticity, oscillatory activity, and network resilience (Beard et al., 2021; Castro and Potente, 2022).

A systems perspective further highlights the bidirectional coupling between peripheral physiology and CNS function. Hormonal signals, circulating lipids, amino acids, and metabolic intermediates may link whole-body energy homeostasis with brain activity (Beltran-Velasco and Clemente-Suárez, 2025; Müller et al., 2025a,c). Nutritional imbalance–whether through chronic caloric excess, micronutrient deficiency, or altered metabolic flexibility–can shift this integrated network toward maladaptive states characterized by mitochondrial dysfunction, chronic low-grade inflammation, and impaired proteostasis. Such conditions may closely mirror the biological hallmarks of brain aging and neurodegeneration (Charbit et al., 2025; Fontana et al., 2021; Zong et al., 2024).

Importantly, nutritional signaling operates across multiple temporal scales. Acute nutrient fluctuations can rapidly influence neuronal excitability and neurotransmitter synthesis, whereas long-term dietary patterns may contribute to remodeling of epigenetic landscapes that stabilize cellular phenotypes over years or decades (Gómez-Pinilla, 2008; Ostaiza-Cardenas et al., 2025). While much of the causal evidence for such long-term epigenetic effects derives from experimental and animal studies, emerging human data suggest that sustained dietary exposures can be associated with persistent epigenetic signatures. Examples include genome-wide DNA methylation changes linked to habitual dietary patterns (Ma et al., 2020; Mandaviya et al., 2019) and long-lasting DNA methylation differences following prenatal famine exposure (Heijmans et al., 2008). This temporal layering provides a potential mechanistic framework for understanding how lifelong nutritional environments may shape brain resilience and vulnerability across the aging trajectory.

Within this systems-level framework, nutrition can be conceptualized as a regulatory interface connecting metabolism, immunity, and neural plasticity. Rather than acting as a single therapeutic factor, nutritional modulation influences distributed biological networks that may collectively determine brain vulnerability or resilience. The following sections examine how these principles manifest across key mechanistic domains, beginning with nutrient-sensing pathways that preserve synaptic integrity and proteostatic balance in the aging brain.

## 3 Nutrient-sensing pathways preserving synaptic integrity and proteostasis

**FIGURE 1:**
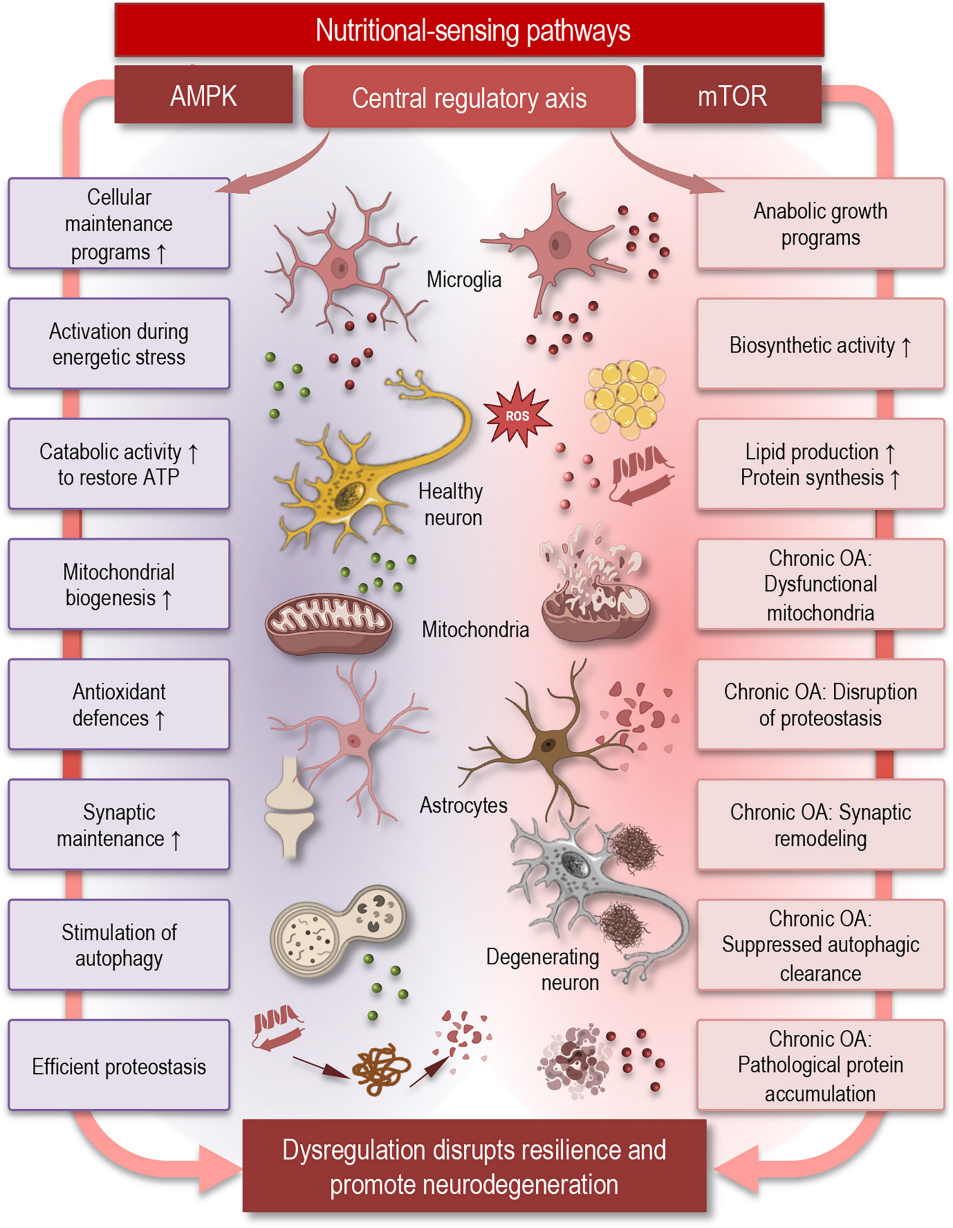
Nutrient-sensing pathways regulating cellular maintenance and neurodegeneration. Schematic overview of the central nutrient-sensing regulatory axis controlling cellular metabolism and proteostasis in the brain. The balance between AMPK (left) and mTOR (right) signaling coordinates adaptive responses to cellular energy status. AMPK activation during energetic stress promotes catabolic pathways that restore ATP levels and support cellular maintenance, including mitochondrial biogenesis, antioxidant defenses, synaptic maintenance, stimulation of autophagy, and efficient proteostasis. In contrast, mTOR signaling promotes anabolic growth programs and biosynthetic activity, including lipid production and protein synthesis. When nutrient-sensing balance is disrupted, excessive anabolic signaling may contribute to mitochondrial dysfunction, impaired proteostasis, suppressed autophagic clearance, and pathological protein accumulation. These processes influence neurons and glial cells–including microglia and astrocytes–affecting mitochondrial function, oxidative stress, and synaptic remodeling. The loss of coordinated regulation across these pathways can shift cellular states from homeostasis toward neurodegeneration, reducing resilience and promoting neuronal damage during aging. OA, overactivation; AMPK, AMP-activated protein kinase; mTOR, mammalian target of rapamycin; ATP, adenosine triphosphate.

The ability of the brain to maintain functional stability despite continuous metabolic and environmental challenges depends on tightly regulated nutrient-sensing pathways that coordinate cellular energy status with adaptive responses (Figure 1). Rather than acting solely as metabolic fuels, nutrients activate conserved signaling networks that regulate protein turnover, mitochondrial function, stress resistance, and synaptic plasticity–processes fundamentally linked to brain aging and neurodegeneration.

AMP-activated protein kinase–mTOR antagonism represents a central regulatory axis controlling the balance between biosynthetic activity and cellular maintenance programs (Figure 1). These systems collectively integrate information about nutrient availability, redox balance, and cellular stress to determine whether cells engage anabolic growth programs or shift toward maintenance and repair (Garza-Lombó et al., 2018; Herzig and Shaw, 2018). In neurons, this balance is particularly critical because synaptic transmission imposes exceptionally high energetic demands while limiting opportunities for cellular renewal. Age-associated shifts toward persistent mTOR activity and reduced AMPK responsiveness contribute to impaired autophagic flux, accumulation of damaged organelles, and reduced stress tolerance (Garza-Lombó et al., 2018). These changes are increasingly recognized as shared mechanistic features linking immunosenescence, chronic low-grade inflammation, and neurodegenerative vulnerability.

AMP-activated protein kinase functions as a metabolic checkpoint activated during energetic stress, promoting catabolic pathways that restore ATP levels and stimulate autophagy (Figure 1). Activation of AMPK enhances mitochondrial biogenesis, antioxidant defenses, and synaptic maintenance, thereby supporting neuronal resilience under conditions of metabolic challenge. In contrast, mTOR signaling promotes protein synthesis, lipid production, and synaptic remodeling during nutrient abundance (Garza-Lombó et al., 2018; Herzig and Shaw, 2018; Vergara Nieto et al., 2025). While physiological mTOR activity is required for learning and memory, chronic overactivation may disrupt proteostasis, suppress autophagic clearance, and contribute to pathological protein accumulation observed in neurodegenerative disorders (Raab-Graham and Niere, 2017).

Proteostasis–the dynamic balance between protein synthesis, folding, trafficking, and degradation–represents a critical convergence point between nutritional signaling and neurodegeneration. Neurons rely heavily on efficient proteostatic mechanisms due to their longevity and complex morphology. Nutrient-dependent regulation of autophagy and lysosomal pathways enables the removal of damaged organelles and misfolded proteins, preventing toxic aggregation and preserving synaptic function (Boucher et al., 2025; Garza-Lombó et al., 2018; Lin et al., 2023; Shukla and Narayan, 2025; Tamatta et al., 2025). Age-associated impairments in these pathways are increasingly viewed as early drivers of disorders such as AD and PD.

Beyond intracellular quality control, nutrient sensing may also shape synaptic resilience at the circuit level. Dietary states may influence neurotransmitter synthesis, vesicle recycling, dendritic spine remodeling, and local protein translation within synapses. Metabolic flexibility–the capacity to switch between glucose, fatty acids, and ketone bodies–can support sustained neuronal activity and protect against energetic failure (Di Lucente et al., 2024; Fadó et al., 2022).

Importantly, nutrient-sensing pathways operate within interconnected cellular networks involving neurons, astrocytes, and microglia. Astrocytes can regulate metabolic substrate delivery and redox buffering, whereas microglial metabolic programs may influence immune surveillance and inflammatory tone. Nutritional signals thus propagate through neuroglial interactions, coordinating cellular maintenance programs that sustain network stability (Hayashide et al., 2026; Zhou and Jiang, 2025).

Collectively, these observations support a model in which nutrient-sensing pathways may function as master regulators linking metabolic status to synaptic integrity and long-term brain resilience. Dysregulation of these signaling systems may represent a unifying mechanism connecting aging, metabolic imbalance, and neurodegenerative disease vulnerability. The following section examines how bioenergetic and membrane-associated processes further translate nutritional signals into changes in neuronal excitability and network dynamics.

## 4 Bioenergetic and membrane processes governing neuronal excitability and network stability

Neuronal excitability and network stability critically depend on energy availability and membrane integrity. Neurons rely on high rates of ATP production to sustain ionic gradients, neurotransmitter cycling, vesicular trafficking, and ensure efficient removal of dysfunctional organelles (Fadó et al., 2022). Perturbations in bioenergetics can therefore compromise synaptic transmission and circuit function, contributing to cognitive decline during aging (Grimm, 2021).

Mitochondrial dynamics, including fusion, fission, and mitophagy, are central to maintaining localized energy supply at synapses (Chen and Chan, 2009). Nutrient-sensitive pathways can modulate these processes: AMPK activation promotes mitochondrial quality control, whereas mTORC1 signaling supports dendritic growth and synaptic remodeling under nutrient-rich conditions (Goul et al., 2023; Jiang, 2024). Lipid metabolism can also influence membrane composition, affecting ion channel function, receptor localization, and vesicle fusion efficiency - linking diet-derived metabolites to neuronal excitability (Incontro et al., 2025).

Bioenergetic flexibility–the capacity to switch between glucose, fatty acids, and ketone bodies–can enhance resilience under metabolic stress. Ketone bodies, for example, may provide an alternative fuel while modulating neuronal redox state and neurotransmission. Disruptions in these processes, as observed in aging or neurodegenerative diseases, can lead to synaptic failure, network instability, and progressive neuronal vulnerability observed in aging and neurodegenerative disease (Jensen et al., 2020; Na et al., 2025).

At the systems level, bioenergetic and membrane mechanisms integrate with glial support: astrocytes buffer extracellular potassium, supply metabolic substrates, and regulate neurotransmitter uptake, while oligodendrocytes influence axonal conduction through myelin maintenance. These coordinated cellular interactions may translate nutritional signals into stable circuit function (Grimm, 2021; Lee et al., 2022; Philips and Rothstein, 2017). Importantly, metabolic signals can also shape glial activation states and inflammatory responses, linking cellular energy status to neuroinflammatory cascades in the aging brain.

## 5 Metabolic regulation of glial activation states and neuroinflammatory cascades

Glial cells represent central regulators of immune homeostasis within the CNS, and their functional phenotypes are closely linked to cellular metabolism. Astrocytes, microglia, and oligodendrocyte lineage cells dynamically adjust metabolic programs in response to nutrient availability, neuronal activity, and cellular stress. Increasing evidence suggests that metabolic flexibility may influence whether glia adopt neuroprotective or pro-inflammatory states (Deczkowska et al., 2018; Pang et al., 2026).

Microglia demonstrate pronounced immunometabolic plasticity. Under homeostatic conditions, microglia primarily rely on mitochondrial oxidative phosphorylation, which is associated with immune surveillance and tissue maintenance. During injury, aging, or metabolic stress, microglia can undergo metabolic reprogramming toward glycolysis accompanied by altered mitochondrial dynamics and inflammatory signaling. Such metabolic transitions may promote cytokine production and oxidative stress responses that, when sustained, can contribute to synaptic dysfunction and neurodegenerative processes (Lauro and Limatola, 2020; Pang et al., 2026).

Astrocytes function as key metabolic integrators linking systemic energy status with neuronal activity. By regulating glucose uptake, glycogen storage, lactate supply, and neurotransmitter recycling, astrocytes support neuronal metabolism and help maintain excitatory–inhibitory balance. Changes in astrocytic bioenergetics may influence redox homeostasis, neurotransmitter clearance, and network stability, suggesting that glial metabolism can directly affect circuit function (Beard et al., 2021; Lee et al., 2022; Pang et al., 2026).

Oligodendrocytes are similarly metabolically sensitive due to the high energetic demands of myelin synthesis and maintenance. Disturbances in lipid metabolism or mitochondrial function may impair myelin integrity and axonal support, potentially contributing to cognitive decline observed during aging and neurodegeneration (Philips and Rothstein, 2017).

Nutrient-sensing signaling pathways integrating cellular energy status can modulate glial autophagy, mitochondrial quality control, and inflammatory responses. Dysregulation of these pathways may amplify neuroinflammatory cascades, disrupt proteostasis, and lower resilience to neurodegenerative stressors (Müller and Di Benedetto, 2026; Ondaro et al., 2022; Tamatta et al., 2025; Yan et al., 2025). Importantly, glial activation states appear to reflect systemic metabolic conditions, as circulating metabolites, hormonal signals, and diet-derived molecules can influence neuroimmune tone (Nampoothiri et al., 2022).

Conversely, metabolic interventions–including caloric moderation, intermittent fasting, or ketogenic metabolic states–have been shown in experimental models to promote adaptive glial phenotypes, characterized by enhanced mitochondrial efficiency and attenuated inflammatory signaling. While much of the evidence remains preclinical or observational, these findings support the idea that modulation of glial metabolism may serve as a mechanistic link between nutritional status and CNS immune regulation, providing a rationale for future translational studies (Alkurd et al., 2024; Cuevas-Martínez et al., 2024; Di Lucente et al., 2024; Dyńka et al., 2022; Rahmani et al., 2023; Spadaro et al., 2022; Vergara Nieto et al., 2025; Yang and Zhang, 2020; Zhang et al., 2024).

Taken together, glial metabolism represents a critical interface through which nutritional processes may influence CNS immunity. By linking systemic metabolic state with local inflammatory regulation, glial immunometabolism provides a mechanistic bridge connecting diet, aging biology, and neurodegenerative disease progression.

## 6 Gut-derived metabolites modulating BBB dynamics, immune crosstalk, and CNS epigenetics

Microbiota-derived metabolites are increasingly recognized as important modulators of brain physiology, linking intestinal nutrient processing with neural and immune regulation (Müller and Di Benedetto, 2025a,^2026^; Vergara Nieto et al., 2025). Diet-dependent microbial metabolism generates a diverse spectrum of small molecules capable of influencing endothelial function, peripheral immune signaling, and cellular responses within the brain. Among these, short-chain fatty acids (SCFAs) such as butyrate and propionate have been associated with maintenance of BBB stability and vascular homeostasis. Experimental studies suggest that these metabolites may strengthen endothelial barrier properties, regulate transport processes, and modulate inflammatory signaling at the neurovascular interface. Through effects on endothelial metabolism and immune signaling, microbial metabolites may contribute to limiting excessive peripheral immune cell trafficking into neural tissue, thereby supporting controlled neuroimmune communication (Chenghan et al., 2025; Silva et al., 2020).

The microbiome also shapes systemic immune tone, which can indirectly influence BBB permeability and neuroinflammatory susceptibility. Age-related alterations in microbial composition have been associated with increased circulating inflammatory mediators that may weaken barrier regulation and promote chronic low-grade inflammation. In this context, the BBB emerges not as a static physical boundary but as a metabolically responsive interface integrating nutritional, microbial, and immune-derived signals (Chenghan et al., 2025; Conway and Duggal, 2021; Huang et al., 2023; Müller and Di Benedetto, 2025b).

Beyond vascular regulation, microbial metabolites such as tryptophan derivatives, bile acids, and phenolic compounds may influence neural function through metabolic and epigenetic pathways. Several of these molecules interact with nuclear receptors and chromatin-modifying enzymes, potentially altering transcriptional programs in neurons and glial cells. By modulating epigenetic regulatory mechanisms, microbiome-derived signals may contribute to long-term adaptations in neuroimmune responsiveness and cellular stress resilience. Nutrient-driven shifts in microbial metabolism or dysbiosis may therefore propagate inflammatory signaling and epigenetic alterations associated with cognitive aging and neurodegenerative vulnerability (Dalile et al., 2019; Marrocco et al., 2020; McGee et al., 2024; Perlmutter et al., 2024).

Importantly, gut–brain communication appears to operate bidirectionally. Neural activity, stress responses, and systemic metabolic states can influence gastrointestinal physiology, microbial community structure, and metabolite production. This reciprocal regulation suggests that brain aging may both influence and be influenced by microbiome dynamics (Müller and Di Benedetto, 2025a,^2026^). Conceptualizing the gut–brain axis as an adaptive signaling network highlights the possibility that dietary modulation and microbiome-directed strategies may support brain resilience, although causal mechanisms in humans remain incompletely defined.

## 7 Epigenetic reprogramming and brain aging trajectories

Aging of the CNS is increasingly understood as a process shaped not only by genetic predisposition but also by dynamic epigenetic regulation. Epigenetic mechanisms–including DNA methylation, histone modifications, chromatin remodeling, and non-coding RNA signaling–may translate environmental and metabolic inputs into long-lasting changes in gene expression without altering DNA sequence. Nutrition can represent one of the most powerful modulators of these processes, linking metabolic state to neuronal function and brain aging trajectories (Bekdash, 2024).

Neurons and glial cells exhibit distinct yet interconnected epigenetic landscapes that remain plastic throughout life. Age-associated epigenetic drift, characterized by altered DNA methylation patterns, reduced chromatin stability, and impaired transcriptional precision, may contribute to synaptic dysfunction, mitochondrial decline, and increased neuroinflammatory susceptibility. Nutrient availability can directly influence these processes by regulating the intracellular pools of metabolic cofactors required for epigenetic enzymes. Metabolites such as acetyl-CoA, S-adenosylmethionine, α-ketoglutarate, flavin adenine dinucleotide, and NAD^+^ serve as essential substrates or regulators of histone acetyltransferases, DNA methyltransferases, demethylases, and sirtuin deacetylases (Bekdash, 2024; Sen et al., 2016). Consequently, cellular metabolism and epigenetic control can form an integrated regulatory system.

Dietary patterns capable of modifying systemic metabolism have been shown to influence chromatin accessibility and transcriptional programs associated with stress resistance and neuronal maintenance. Activation of NAD^+^-dependent signaling pathways can promote genomic stability, enhance mitochondrial function, and support adaptive responses to oxidative and inflammatory stress. Conversely, chronic nutrient excess and metabolic dysregulation may accelerate epigenetic aging signatures that parallel cognitive decline and increased vulnerability to neurodegenerative disease (Covarrubias et al., 2021; Horvath and Raj, 2018; Jensen et al., 2020; Missong et al., 2024; Spadaro et al., 2022).

Epigenetic regulation may also represent a key interface between peripheral physiology and CNS resilience. Immune-derived signals, hormonal cues, and microbiota-associated metabolites can induce chromatin remodeling in microglia and astrocytes, thereby shaping long-term inflammatory set points within neural circuits. These processes suggest that brain aging may emerge from cumulative epigenetic adaptations to lifelong metabolic exposure rather than from irreversible neuronal loss alone (Bekdash, 2024; Giallongo et al., 2022; Li et al., 2023).

Importantly, epigenetic modifications retain a degree of reversibility. Emerging evidence indicates that metabolic and nutritional interventions may partially restore adaptive transcriptional programs, improving synaptic plasticity and cognitive performance even later in life. Within this framework, nutrition may function as a continuous epigenetic signal capable of modulating the trajectory of brain aging, shifting biological systems toward either degeneration or resilience (Ostaiza-Cardenas et al., 2025; Sen et al., 2016; Singh et al., 2024).

## 8 Systems integration: nutrition–microbiome–epigenome convergence in brain aging

**FIGURE 2:**
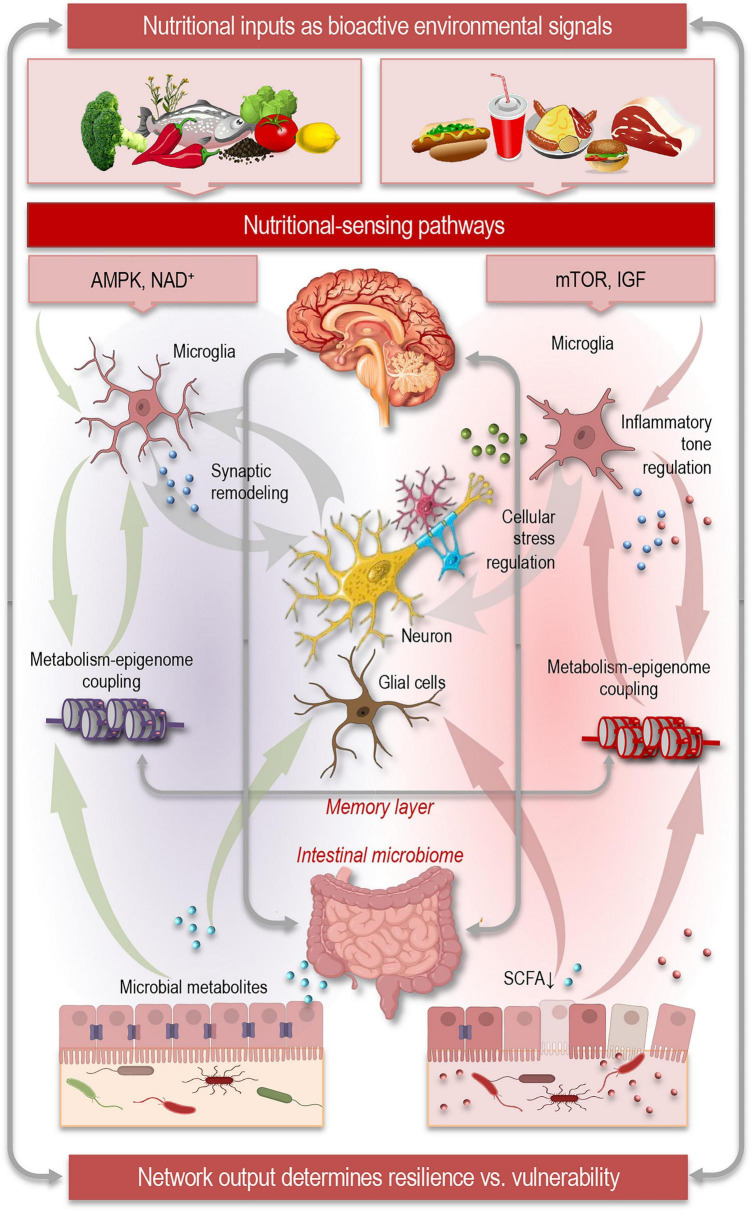
Systems integration of nutrition, microbiome, and epigenome in brain aging. Brain aging may emerge from interactions across multiple regulatory layers linking environmental inputs to neural network function. Nutrition represents the input layer, providing biochemical signals that are processed by host metabolism and the intestinal microbiome, which acts as an intermediate signaling hub generating bioactive metabolites. These signals engage nutrient-sensing pathways and metabolic programs in neurons and glial cells. In parallel, epigenetic mechanisms function as a memory layer, stabilizing transcriptional responses to metabolic and microbial signals over time. Glial cells and neuroimmune networks serve as integrative effectors where metabolic, microbial, and immune signals converge to regulate inflammatory tone, synaptic remodeling, and cellular stress responses. Across these regulatory layers–nutrition, microbiome metabolism, nutrient sensing, epigenetic memory, and neuroimmune signaling–the emergent network output may determine resilience versus vulnerability during brain aging, influencing circuit stability, cognitive trajectories, and susceptibility to neurodegenerative disease. AMPK, AMP-activated protein kinase; mTOR, mammalian target of rapamycin; IGF, insulin/IGF signaling pathways; NAD^+^, nicotinamide adenine dinucleotide dependent sirtuins; SCFA, short-chain fatty acids.

Building on the mechanistic insights discussed above, an integrative perspective is required to understand how nutritional signals, microbial metabolism, and epigenetic regulation may collectively shape neuroimmune networks and ultimately influence trajectories of brain aging (Figure 2). Dietary components represent continuous environmental signals translated into biochemical information through host metabolism and microbial transformation (Mazzio and Soliman, 2014). From a systems perspective, nutrition therefore establishes the input layer of brain aging regulation, influencing downstream cellular states long before structural neurodegeneration becomes apparent.

Brain aging and resilience may emerge from the dynamic interplay of multiple regulatory layers, in which nutrition functions as a system-level regulator of neural and glial function. Dietary inputs act not merely as energy sources but as bioactive signals that can reprogram cellular metabolism, modulate neuroimmune networks, and influence epigenetic landscapes. Nutrient-sensing pathways in glia and neurons can link systemic metabolic status to synaptic maintenance, proteostasis, and cellular stress responses, mechanisms that may collectively shape the pace and quality of brain aging (Müller and Di Benedetto, 2026; Pignatti et al., 2020).

The intestinal microbiome functions as a dynamic interface translating dietary patterns into bioactive molecular signals and may serve as an intermediate signaling hub. Microbial metabolites–including SCFAs, tryptophan derivatives, and bile acids–may also interact with epigenetic regulators, bridging peripheral nutritional cues with transcriptional programs in CNS cells (Figure 2). This metabolism–epigenome coupling presumably establishes a molecular memory, where metabolic states induced by diet or microbiome activity become encoded as durable transcriptional programs. This positions epigenetic regulation as the memory layer of environmental exposure, that can influence the trajectory of neuronal and glial aging, potentially preserving adaptive phenotypes or, under dysregulated conditions, promoting vulnerability to age-related cognitive decline (Li et al., 2022, 2023; Stolberg, 2025).

Glial cells may represent critical convergence points where metabolic, microbial, and epigenetic signals intersect. Microglia and astrocytes continuously sense peripheral immune signals and metabolic cues, adapting their activation states along a continuum from neuroprotective to neurotoxic phenotypes (Müller and Di Benedetto, 2026; Seguin et al., 2023; Stolberg, 2025). Neuroimmune networks act as integrative effectors, receiving convergent signals from metabolism, microbial metabolites, and epigenetic mechanisms to calibrate inflammatory tone, synaptic remodeling, and repair capacity. The emergent output of this network may determine resilience versus vulnerability during aging (Figure 2): when regulatory systems remain balanced, circuits can maintain plasticity and functional stability; when dysregulated, metabolic inflexibility, microbial imbalance, or epigenetic rigidity may propagate chronic neuroinflammation and synaptic dysfunction, accelerating neurodegenerative risk and cognitive decline (Müller et al., 2025a; Seguin et al., 2023).

Integrating these interacting regulatory layers supports a model in which brain aging reflects the cumulative stability of interconnected biological networks rather than isolated molecular damage (Müller and Di Benedetto, 2026; Müller et al., 2025a). Nutritional inputs act as programmable environmental signals that shape organismal physiology at multiple hierarchical levels. The microbiome transforms dietary exposures into metabolite-based signaling cues that influence systemic metabolism and immune activity. These metabolic signals determine cellular functional states, while epigenetic mechanisms stabilize adaptive or maladaptive transcriptional programs over time. Neuroimmune networks ultimately translate these integrated signals into functional outcomes affecting synaptic maintenance, neuronal survival, and circuit stability.

Within this systems framework, healthy aging can be conceptualized as the preservation of network flexibility–the capacity of neural systems to dynamically recalibrate responses to metabolic stress, inflammatory challenges, and environmental change. In contrast, neurodegenerative disease may emerge when regulatory networks progressively lose adaptive plasticity and become locked into self-reinforcing states characterized by chronic inflammation, impaired bioenergetics, and reduced repair capacity (Müller and Di Benedetto, 2026; Müller et al., 2025a).

Overall, this framework suggests that brain aging can be viewed as an emergent property of multi-layered signaling interactions, in which nutrition, microbiome dynamics, metabolism, epigenetic regulation, and neuroimmune signaling converge to shape individual aging trajectories. By identifying points where these signals may be modulated, this model provides a mechanistic foundation for strategies aimed at preserving network stability, cognitive function, and adaptive brain resilience across the lifespan.

## 9 Translational outlook: leveraging nutritional signals to brain resilience

The convergence of nutritional signaling, microbiome activity, metabolic regulation, and epigenetic plasticity suggests that brain aging may be modifiable through biologically informed interventions targeting system-level regulation rather than isolated disease pathways. Nutrition may therefore represent a tractable entry point for translational neuroscience because dietary exposures act continuously across the lifespan and influence multiple interacting mechanisms underlying neurodegeneration (Charbit et al., 2025; Müller and Di Benedetto, 2025b; Park et al., 2025).

Viewing brain aging through this integrated nutrition–microbiome–epigenome axis may shift therapeutic thinking from late-stage intervention toward early systems modulation. Clinical translation should also incorporate lifestyle and dietary patterns known to enhance metabolic flexibility, such as caloric moderation, intermittent fasting, or ketogenic diet, which can promote adaptive glial phenotypes and reduce chronic neuroinflammatory signaling. Lifestyle-associated factors including diet quality, metabolic health, circadian regulation, and physical activity may interact synergistically to influence biological aging trajectories long before clinical symptoms arise (Dyńka et al., 2022; Fekete et al., 2025; Fontana et al., 2021; Müller and Di Benedetto, 2025a, c; Nassan and Videnovic, 2022; Wang and Li, 2021). Preventive strategies therefore increasingly emphasize maintaining systemic metabolic balance and neuroimmune stability across the lifespan.

Interindividual variability represents a major challenge in translating advances in nutritional neuroscience into effective clinical strategies. Genetic background, biological sex, microbiome composition, metabolic health status, and lifelong environmental exposures collectively shape how individuals respond to dietary interventions (Healey et al., 2017; Mansour et al., 2024; Müller et al., 2025b; Nguyen et al., 2025). Consequently, identical nutritional approaches may produce markedly different neurobiological outcomes across populations. Rather than prescribing uniform dietary recommendations, individualized strategies may optimize brain aging trajectories according to biological context (Mansour et al., 2024).

Emerging precision medicine frameworks aim to address this variability by integrating multidimensional biological profiling. Metabolic phenotyping, including assessments of insulin sensitivity and systemic energy regulation, provides insight into individual metabolic resilience. Microbiome-derived metabolomic signatures offer additional information regarding host–microbial interactions influencing neuroimmune signaling. Epigenetic biomarkers reflecting biological aging processes, together with neuroimaging indicators of network integrity and cognitive reserve, further contribute to individualized risk stratification (Li and Qi, 2022; Mansour et al., 2024; Qi, 2022; Singh et al., 2024). Incorporating behavioral and lifestyle data alongside these biological measures may ultimately allow the development of personalized nutritional strategies tailored to support neuronal bioenergetics, maintain balanced neuroimmune responses, and preserve synaptic plasticity across the lifespan.

Precision nutrition approaches that integrate multidimensional biological profiling can potentially identify individuals most likely to benefit from specific dietary or microbiome-targeted interventions (Bianchetti et al., 2023). For example, metabolomic and microbiome signatures may guide supplementation with SCFAs, tryptophan derivatives, or other bioactive nutrients to support BBB integrity, neuroimmune balance, and synaptic plasticity (Chenghan et al., 2025; Farhadipour et al., 2023; Nagpal et al., 2019). Epigenetic readouts may further enable monitoring of long-term adaptive responses to these interventions, providing mechanistic insight into their efficacy (Bekdash, 2024; Burdusel et al., 2024; Li et al., 2023).

Advances in digital health and systems medicine may enable continuous integration of dietary behavior with physiological monitoring. Wearable technologies, metabolomics platforms, and machine-learning–based nutritional modeling are beginning to support adaptive intervention strategies that dynamically respond to individual biological states rather than static dietary recommendations. Such approaches may allow real-time adjustment of nutritional exposures to maintain metabolic and neuroimmune stability (Bianchetti et al., 2023; Mortazavi and Gutierrez-Osuna, 2023; Shi et al., 2022; Wang et al., 2022; Zahedani et al., 2023).

Nutritional interventions may function as metabolic neuromodulators capable of reshaping neuroimmune tone and neuronal bioenergetics. Dietary modulation of lipid metabolism, micronutrient availability, and mitochondrial substrates can influence inflammatory signaling and synaptic function, suggesting potential roles as adjunctive therapies in neurodegenerative disease. Importantly, translational success will likely depend on early implementation during preclinical or prodromal stages of brain aging, when adaptive plasticity remains preserved (Davì et al., 2025; Dyńka et al., 2022; Fang et al., 2026; Jiang et al., 2022).

In addition, diet-derived metabolites capable of influencing chromatin regulation provide a potential avenue for reshaping long-term transcriptional programs associated with aging and neurodegeneration. Modulation of the gut microbiome through dietary composition, fiber intake, and fermentation-derived nutrients may further restore disrupted gut–brain communication pathways. Importantly, these intervention domains should be understood as interdependent components of a unified systems framework rather than independent therapeutic strategies (Bourassa et al., 2016; Marín-Tello et al., 2022; Nguyen et al., 2025).

Finally, translation requires moving beyond symptom management toward resilience-oriented prevention paradigms. Nutritional signaling may help stabilize network-level brain function, preserve BBB integrity, and maintain epigenetic flexibility that supports healthy aging trajectories. Large-scale longitudinal trials combining nutrition, lifestyle, and systems-biology biomarkers will be essential to determine whether nutritional modulation can delay or reduce the burden of AD, PD, and age-associated cognitive decline (Bauer and Thiele, 2018; Charbit et al., 2025; Fontana et al., 2021; Mansour et al., 2024; Park et al., 2025).

Taken together, the emerging evidence suggests that nutrition can be conceptualized as a programmable biological signal capable of influencing brain aging across molecular, cellular, and systems levels. Integrating mechanistic neuroscience with clinical nutrition science may therefore open a realistic pathway toward preventive neurology grounded in biological resilience rather than late-stage disease intervention.

## 10 Limitations and future directions

Nutrition is increasingly recognized as a multi-layered regulator of brain resilience, yet several critical knowledge gaps remain. First, longitudinal studies integrating multi-omics readouts–metabolomics, epigenomics, transcriptomics, and microbiome profiling–are urgently needed to establish causal links between specific dietary patterns and adaptive neural states over time. Such studies should incorporate age, sex, genetic background, and lifestyle factors to resolve interindividual variability in response to nutritional interventions (Bilgel et al., 2023; Charbit et al., 2025; Dhana et al., 2024; Fekete et al., 2025; Xu et al., 2024).

While preclinical studies have been instrumental in elucidating mechanistic links between nutrition, metabolism, and neuroimmune function, their translational applicability is constrained by several limitations. Animal models are typically characterized by genetic homogeneity, controlled environments, and short lifespans that do not capture the complexity and diversity of human physiology and aging, leading to discrepancies in metabolic and immune responses across species (Frühwein and Paul, 2025). Furthermore, many dietary interventions that produce robust effects in rodents or other models show more modest or inconsistent outcomes in clinical studies of humans, as indicated by systematic comparisons of preclinical and clinical dietary findings. Together, these factors complicate the direct extrapolation of preclinical results to human health, underscoring the need for careful interpretation and targeted translational research (Frühwein and Paul, 2025; Sproten et al., 2024).

Second, mechanistic research must increasingly move beyond associative observations toward causal dissection of how defined nutrient-derived metabolites influence neuronal and glial phenotypes across molecular, cellular, and circuit levels. Advanced experimental platforms now provide unprecedented opportunities to resolve these mechanisms in human-relevant systems (Mrza et al., 2024; Sun and Pan, 2025). Integration of these platforms with single-cell multi-omics, spatial transcriptomics, and metabolomics will likely allow mapping of nutrient–microbiome–brain signaling pathways with cellular resolution. Especially, human induced pluripotent stem cell (iPSC)–derived brain organoids can recapitulate aspects of human cortical development, cellular diversity, and neuroimmune interactions, enabling controlled investigation of metabolic and inflammatory responses within complex multicellular environments (Wenzel et al., 2024). Incorporation of microglia into organoid systems further allows interrogation of neuroimmune–metabolic coupling and disease-associated activation states that are difficult to model in rodents. Complementary humanized microbiome models, may permit causal testing of microbiota-derived metabolites on brain physiology and behavior (Huang et al., 2023; Mir et al., 2026).

Third, deeper integration of computational systems biology and network modeling with experimental datasets will be essential to capture the complexity of nutritional signaling within the brain–body axis. Multiscale modeling approaches capable of linking molecular interactions, cellular phenotypes, and neural circuit dynamics may reveal emergent properties that cannot be inferred from reductionist analyses of isolated pathways (Aminian-Dehkordi et al., 2025; Bauer and Thiele, 2018; De Domenico, 2018; Di Benedetto et al., 2019; Karr et al., 2012; Müller et al., 2025a,d). By integrating metabolic flux analyses, immune signaling networks, microbiome-derived metabolites, and epigenetic regulatory landscapes, such frameworks can uncover critical nodes governing system stability and adaptive capacity. Importantly, these approaches may enable the identification of combinatorial intervention strategies that enhance network robustness, shifting therapeutic design from single-target modulation toward coordinated regulation of interconnected biological systems that sustain brain resilience during aging.

Additionally, translating these mechanistic insights into clinical practice requires development of validated biomarkers capable of tracking brain response to nutritional interventions. Early-phase clinical trials incorporating biomarker-guided dietary modulation, alongside cognitive and neuroimaging outcomes, could pave the way for precision nutrition strategies tailored to individual biological aging trajectories (Talukdar et al., 2023; Zwilling et al., 2024). Finally, translating molecular discoveries into practical dietary recommendations demands interdisciplinary collaboration among neuroscientists, immunologists, metabolic researchers, and clinical practitioners.

## 11 Conclusion

Taken together, current evidence increasingly supports the view that nutrition may act as a central biological signal shaping brain aging trajectories through integrated metabolic, immune, microbial, and epigenetic mechanisms. Rather than acting as passive energetic support, dietary inputs can function as dynamic regulators capable of reprogramming neuronal and glial states across multiple regulatory layers that govern synaptic maintenance, neuroimmune balance, and cellular resilience. Understanding how these regulatory layers interact may enable a shift from reactive neurology toward proactive maintenance of brain resilience.

Viewing brain health through this systems-level framework shifts the paradigm from late-stage disease treatment toward proactive modulation of adaptive capacity throughout the lifespan. Neurodegenerative disease such as AD, PD, and age-associated cognitive decline increasingly appear as outcomes of progressive network dysregulation linking peripheral metabolism with CNS homeostasis. Nutritional modulation therefore may represent a scalable and biologically grounded strategy to reshape brain aging trajectories and to influence brain resilience before irreversible neurodegeneration occurs.

Thus, future progress will depend on integrating molecular neuroscience with precision nutrition, longitudinal biomarker studies, and systems biology approaches capable of capturing individual aging trajectories. By conceptualizing nutrition as an active component of neural regulation, this framework advances a unifying model in which environmental signals can be harnessed to preserve cognitive function and promote healthy brain aging across the lifespan.
